# 3D-printed saw guides for lower arm osteotomy, a comparison between a synthetic CT and CT-based workflow

**DOI:** 10.1186/s41205-021-00103-x

**Published:** 2021-04-29

**Authors:** Koen Willemsen, Mirte H. M. Ketel, Frank Zijlstra, Mateusz C. Florkow, Ruurd J. A. Kuiper, Bart C. H. van der Wal, Harrie Weinans, Behdad Pouran, Freek J. Beekman, Peter R. Seevinck, Ralph J. B. Sakkers

**Affiliations:** 1 Department of Orthopedics, University Medical Center Utrecht, HP:05-228, Heidelberglaan 100, 3584 CX Utrecht, The Netherlands; 2 3D Lab, Division of Surgical Specialties, University Medical Center Utrecht, Utrecht, The Netherlands; 3 Department of Radiology, University Medical Center Utrecht, Utrecht, The Netherlands; 4 Department of Biomechanical Engineering, Delft University of Technology, Delft, The Netherlands; 5 MILabs B.V, Houten, The Netherlands; 6 Department of Translational Neuroscience, Brain Centre Rudolf Magnus, University Medical Centre Utrecht, Utrecht, The Netherlands; 7 Department Radiation Science & Technology, Delft University of Technology, Delft, The Netherlands

## Abstract

**Background:**

Three-dimensional (3D)-printed saw guides are frequently used to optimize osteotomy results and are usually designed based on computed tomography (CT), despite the radiation burden, as radiation-less alternatives like magnetic resonance imaging (MRI) have inferior bone visualization capabilities. This study investigated the usability of MR-based synthetic-CT (sCT), a novel radiation-less bone visualization technique for 3D planning and design of patient-specific saw guides.

**Methods:**

Eight human cadaveric lower arms (mean age: 78y) received MRI and CT scans as well as high-resolution micro-CT. From the MRI scans, sCT were generated using a conditional generative adversarial network. Digital 3D bone surface models based on the sCT and general CT were compared to the surface model from the micro-CT that was used as ground truth for image resolution. From both the sCT and CT digital bone models saw guides were designed and 3D-printed in nylon for one proximal and one distal bone position for each radius and ulna. Six blinded observers placed these saw guides as accurately as possible on dissected bones. The position of each guide was assessed by optical 3D-scanning of each bone with positioned saw guide and compared to the preplanning. Eight placement errors were evaluated: three translational errors (along each axis), three rotational errors (around each axis), a total translation (∆T) and a total rotation error (∆R).

**Results:**

Surface models derived from micro-CT were on average smaller than sCT and CT-based models with average differences of 0.27 ± 0.30 mm for sCT and 0.24 ± 0.12 mm for CT. No statistically significant positioning differences on the bones were found between sCT- and CT-based saw guides for any axis specific translational or rotational errors nor between the ∆T (*p* = .284) and ∆R (*p* = .216). On Bland-Altman plots, the ∆T and ∆R limits of agreement (LoA) were within the inter-observer variability LoA.

**Conclusions:**

This research showed a similar error for sCT and CT digital surface models when comparing to ground truth micro-CT models. Additionally, the saw guide study showed equivalent CT- and sCT-based saw guide placement errors. Therefore, MRI-based synthetic CT is a promising radiation-less alternative to CT for the creation of patient-specific osteotomy surgical saw guides.

## Background

Three-dimensional (3D) preoperative planning and 3D-printed patient-specific implants and saw guides are increasingly used during orthopedic procedures [1–3]. Besides a better understanding of complex anatomies, the use of 3D-printing during surgical procedures can improve surgical results, decrease operating time and decrease radiological exposure [4]. A lower arm osteotomy is one of the orthopedic applications where 3D planning tools and patient-specific saw guides show a significant clinical improvement [5]. For the 3D planning and saw guide design [6, 7], a computed tomography (CT) scan is most commonly used to create a bone model because of its excellent hard tissue contrast and high spatial resolution [8]. However, the CT’s ionizing radiation is harmful, especially for young patients [9]. Even low-dose radiation increases the cancer risk and should be kept as low as possible and alternative procedures should be considered [10, 11].

A radiation-less alternative to CT is Magnetic Resonance Imaging (MRI). MRI-scans generate 3D information without ionizing radiation and provide good quality soft tissue information. Currently, MRI-scans are rarely used for 3D bone modelling and related saw guide design as their lesser bone contrast requires intensive processing to generate 3D bone renderings [12, 13]. Therefore, novel deep learning based models are developed to enhance the bone contrast: MRI-based synthetic CT (sCT) [14]. These deep learning based sCT models use convolutional neural networks (CNN) that translate MRI data into Hounsfield Units (HU). Eventually, with the MRI-scan and simultaneously generated sCT, both good quality soft tissue and hard tissue information is provided with one radiation free acquisition. However, studies investigating sCT for orthopaedic care are scarce and a validation is needed to evaluate the impact of CT-to-sCT differences [15] on 3D digital bone surface modelling and saw guide design.

The primary aim was to investigate whether the sCT-scan provides sufficiently accurate bone surface information for saw guide development when compared to micro-CT (ground truth for image resolution) and how accurate saw guide positioning is when a sCT workflow is used compared to a CT workflow. Therefore, the research question states*: ‘Is the precision of the synthetic-CT, compared to the precision of currently used CT, sufficient for the accurate placement of 3D printed patient-specific lower arm osteotomy saw guides?’* We hypothesized that the sCT-based models would have similar performance to CT-based methods in terms of bone surface modelling and saw guide positioning.

## Materials and methods

### Specimens

**Fig. 1:**
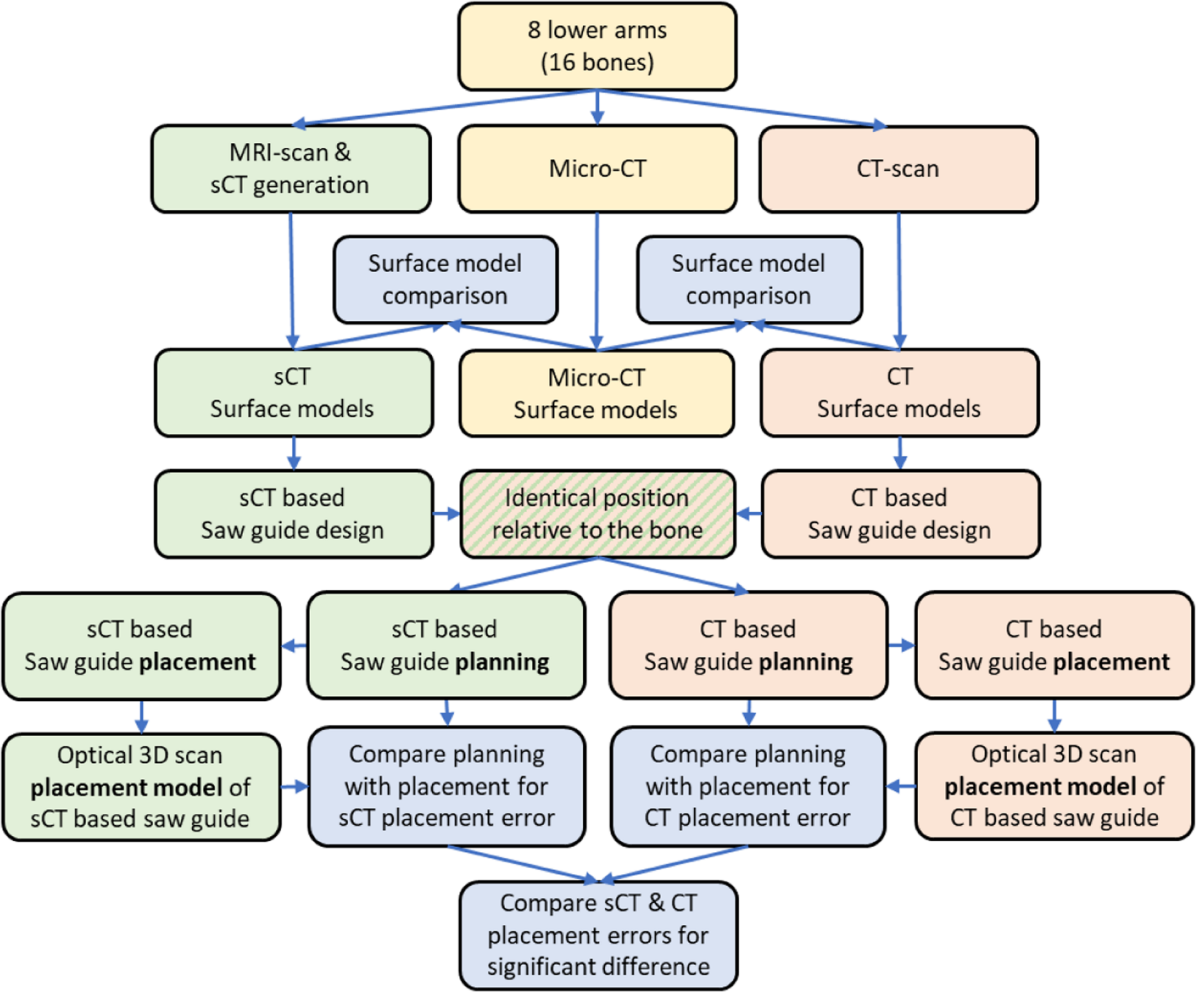
Study overview flowchart. The sCT pipeline is presented in green, the CT pipeline in red and the micro-CT pipeline (ground truth) in yellow. The blue boxes represent the main study outcomes

Eight healthy fresh-frozen human cadaver lower arms (4 left and 4 right, 4 women and 4 men, mean age 78y, ranged 71-86y) were obtained via the Human Body Donation program of the University of Utrecht (Fig. 1).

### Imaging

**Fig. 2:**
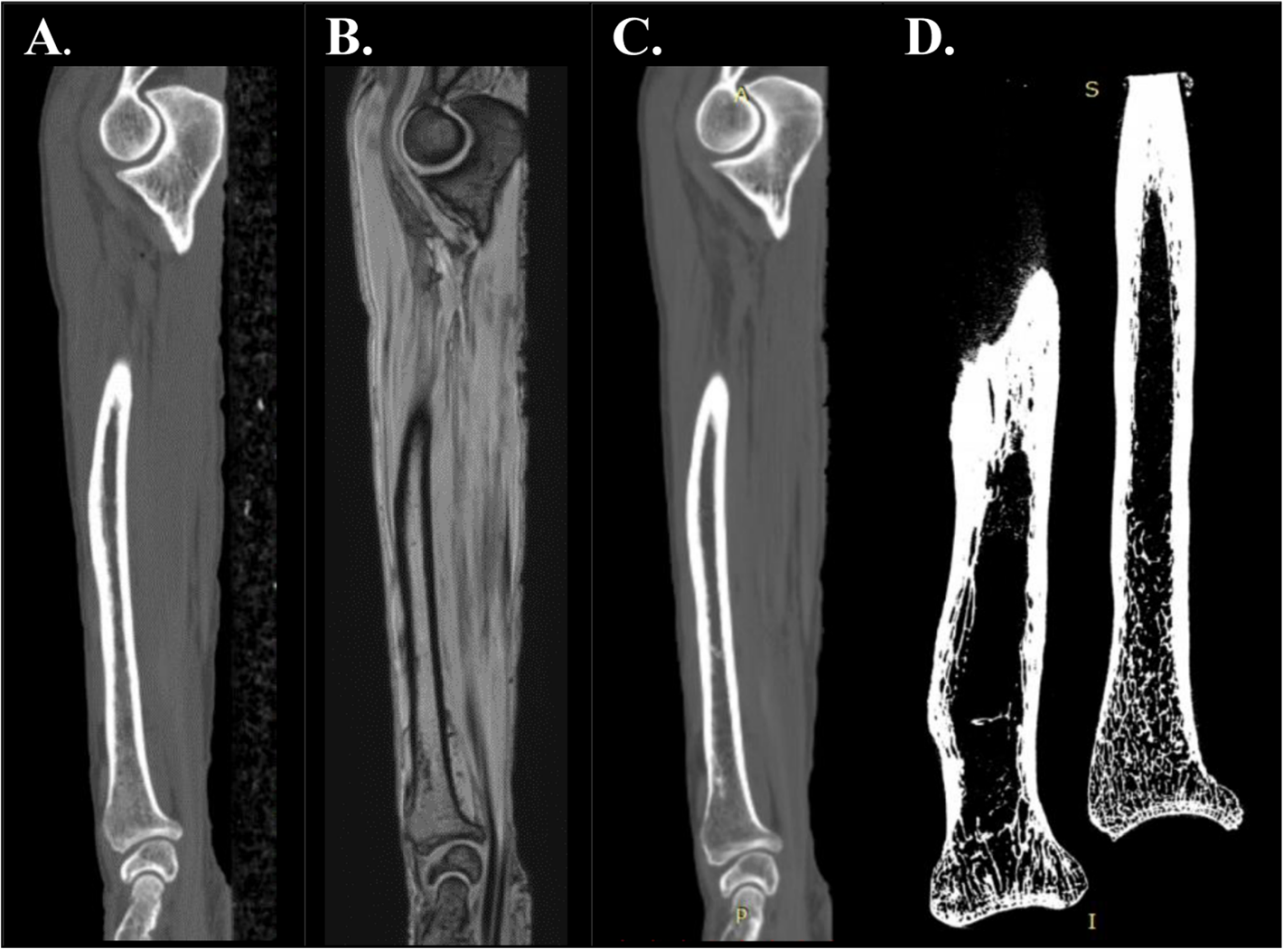
Various imaging modalities from the same left lower arm (P1) **a** Lower arm CT image, **b** Lower arm MRI image, **c** Lower arm sCT image. **d** Micro-CT image of two dissected halves of the same radius bone

With the 24-h thawed intact lower arms fixed in an extended and pronated position, a CT-scan and MRI-scan were acquired in immediately succeeding sessions. The CT-scans (Philips Healthcare, Best, The Netherlands; 120 kV and 250mAs) were obtained with the following parameters: 0.3 × 0.3 mm pixel spacing, 0.8 mm slice thickness and 0.4 mm slice spacing (Fig. 2a). The MRI-images were obtained with a 3 T scanner (Ingenia, Philips Healthcare, Best, The Netherlands) with the following parameters: 1.2 mm isotropic resolution (reconstructed to 0.6 mm), 313x103x128mm field of view, echo times 2.1/3.25/4.4 ms, repetition time 6.9 ms, flip angle 15°, and a total scan duration of 151 s (Fig. 2b).

sCT-scans (Fig. 2c) were generated from the MRI-scans using a 2D conditional generative adversarial network (cGAN) in Python (Python Software Foundation, Wilmington, DE, USA) as previously described by Zijlstra et al. [15]. As ground truth, a 3D micro-CT scan (VECTor6/CT system, MILabs B.V., Utrecht, The Netherlands) was obtained of every bone with the following parameters: multi-circle 360 degrees acquisitions, tuber voltage of 55KV, tube current of 0.19 mA, exposure time of 75 ms per projection, angle increment of 0.5 degrees, and 50 μm reconstructed isotropic voxel size using 3D Feldkamp filtered back-projection reconstruction (Fig. 2d). To fit the arms through the bore and in the micro-CT chamber the bones were cut in half and soft tissue was roughly dissected with standard dissection equipment (i.e. scalpels). After the micro-CT acquisition, the bones were simmered [16] to allow further processing. The influence of simmering on the bone surface was evaluated using a second micro-CT scan (Appendix).

### 3D bone model generation and comparison

**Fig. 3:**
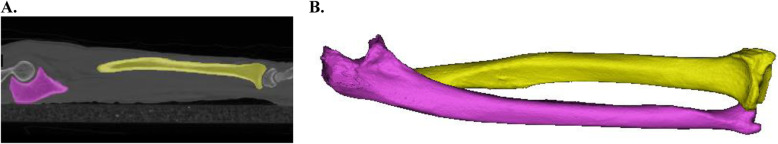
3D-model generation. All scans were segmented (**a**) and the subsequent segmentations were converted in 3D bone models (**b**) in Mimics software. The following settings were used: interpolation method ‘contour’, preferred ‘continuity’, shell reduction to 1, no matrix reduction applied and smoothing factor 0.3 using 2 iterations and exported as binary stereolithography (STL) file. Radius (yellow) and ulna (purple)

A 3D-bone model comparison was performed on semi-automatic bone segmentations of the sCT-, CT- and micro-CT scans generated in Mimics (v21, Materialize NV, Leuven, Belgium). sCT- and CT-segmentations were created based on the thresholding method from Van den Broeck et al. [17] and the micro-CT with Otsu’s [18] automatic thresholding based on Rovaris et al. [19] (Fig. 3). For direct comparison, the generated 3D models were rigidly registered [17]. After registration, the average distances between the 3D model vertices of the ground truth micro-CT and the sCT or CT were calculated in millimeters in 3-matics (v. 13, Materialize NV, Leuven, Belgium). A positive value indicates a larger sCT- or CT-model as compared to the micro-CT model.

### Saw guide generation

**Fig. 4:**
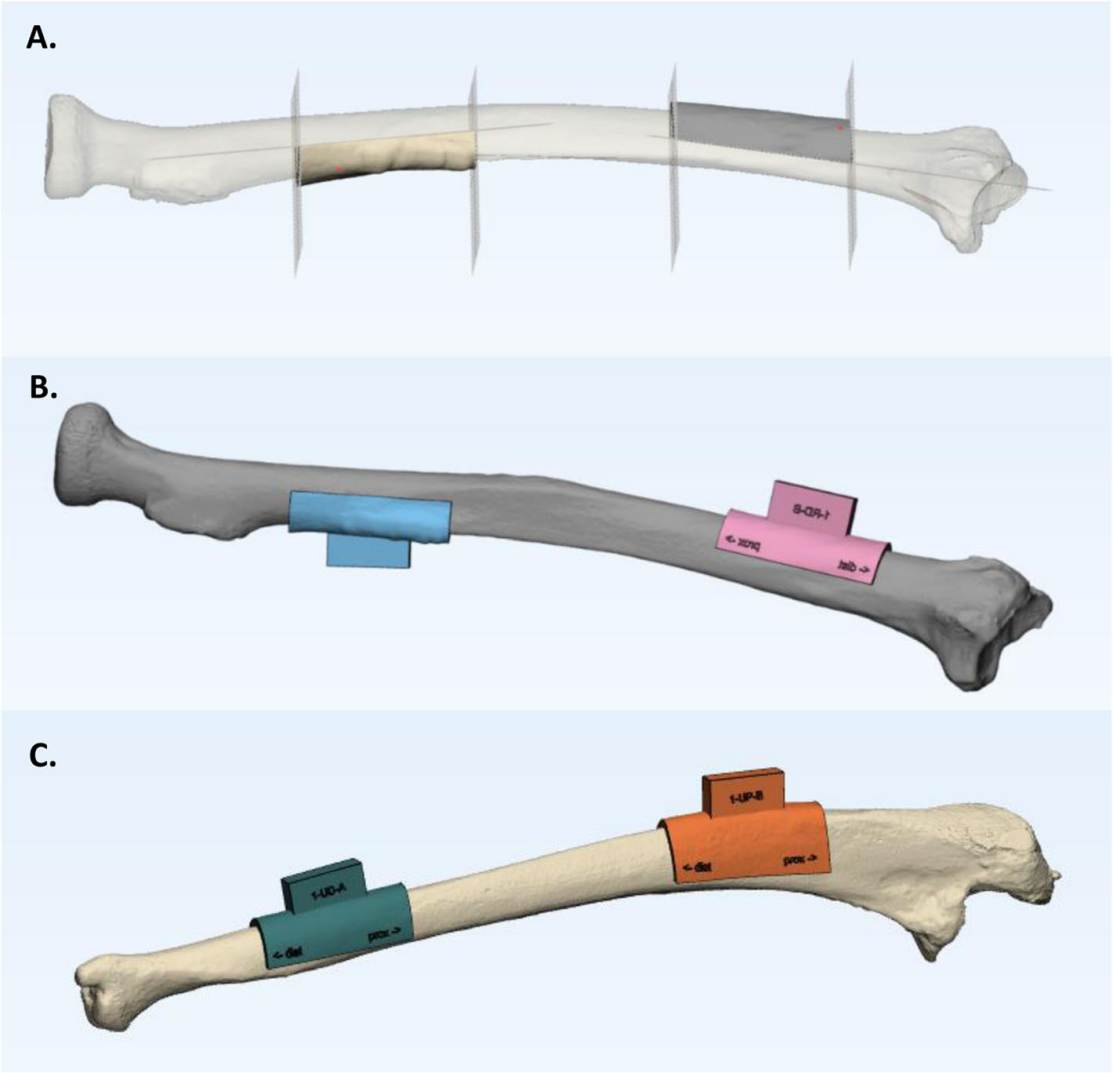
Saw guide generation. **a** A radius CT-3D model with two half cylinders located proximal and distal on the bone, created with the 4 perpendicular and 2 parallel cut-off planes in 3-matic. **b** A radius CT-3D model with its distal (pink) and proximal (blue) saw guide. **c** A ulna CT-3D model with its distal (green) and proximal (orange) saw guides in 3-matic

For all eight radius and ulna bones, one proximal and one distal saw guide was designed per imaging modality, resulting in 32 sCT and 32 CT-based saw guides (Fig. 4). The 40 mm long saw guides [6] were systematically designed (MK) in 3-matic with a reference box (20 × 5 × 10 mm) on top. The relative position to the bone of each saw guide with reference box was identical for both imaging modalities. The 64 saw guides were pseudonymized and 3D-printed using selective laser sintering of nylon powder (PA12) with a printing accuracy 0.12 mm in all directions (P110, EOS, Krailing, Germany). One lateral and one frontal screenshot of the saw guide planning was printed on A4 paper and used as a guide for the observers.

### Observers

Six blinded observers (two orthopedic surgeons, two orthopedic residents in training and two orthopedic researchers) placed the 64 saw guides on the corresponding bone parts, with sCT and CT-based saw guides randomly assigned over two rounds of 32 guides to reduce repetition bias. Observer #3 conducted the study two times with one-week interval, to analyze the intra-observer variability.

### Measuring the position of saw guides

**Fig. 5:**
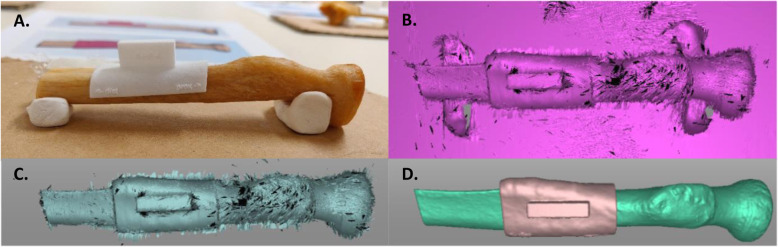
Generation of optical 3D models of a proximal radius bone half and corresponding saw guide after placement. **a** Proximal radius with saw guide after observer placement during the saw guide study. **b** Raw image made by the optical scanner, including the ground, bone holder, bone and saw guide. **c** During post-processing in Artec Studio the regions of interest are selected (saw guide and bone). **d** The final optical scan 3D models of the bone (green) and the saw guide 3D (pink)

To measure the saw guide placement accuracy of each observer, a 0.1 mm voxel size accurate white-light optical 3D scanner (Artec Space Spider, 4C, Emmen, The Netherlands;) was used to scan the position of each saw guide relative to the bone and create a corresponding optical 3D bone with saw guide surface model (Fig. 5).

### Comparing saw guide placement with planning

**Fig. 6:**
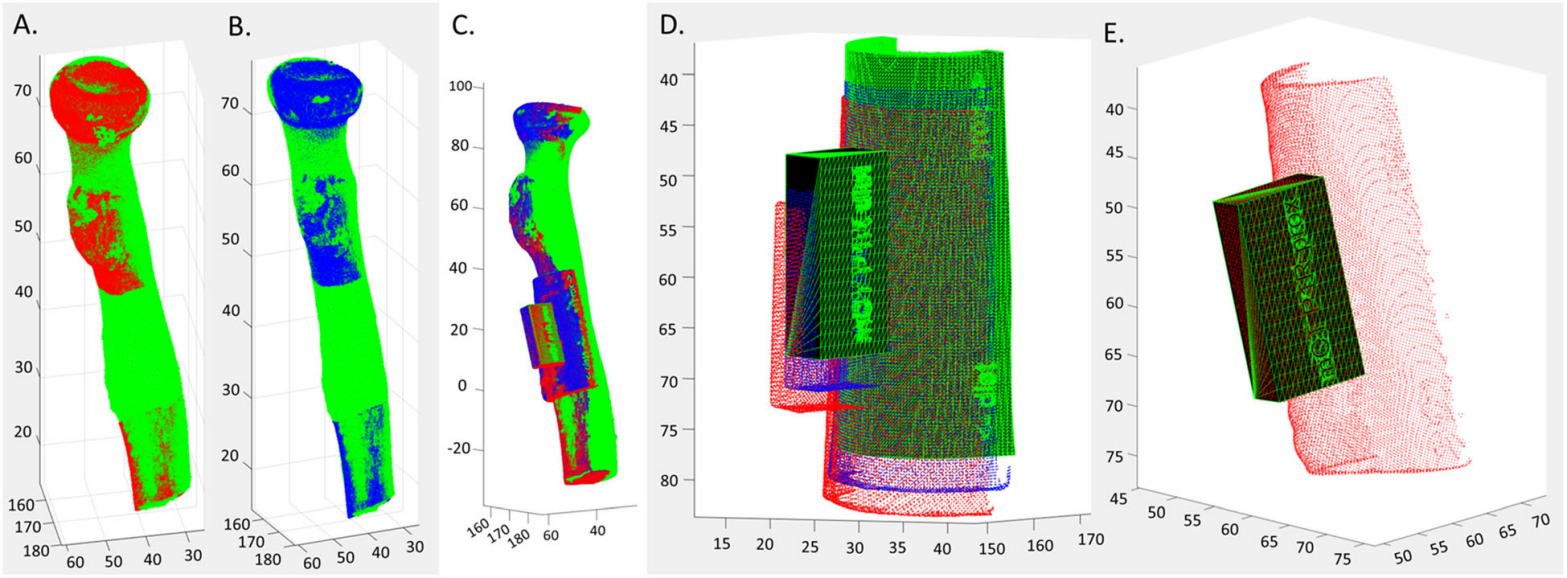
Example calculation of proximal radius saw guide placement error. Registration of the CT (**a** red) and sCT (**b** blue) optical scan bone model (without saw guide) to the micro-CT surface model (green). **c** The registered CT (red) and sCT (blue) optical scan 3D bone models including the relative position of each optical scan’s saw guide model. **d**. The isolated saw guides with their relative positions, revealing the displacements of the CT saw guide (red) and sCT saw guide (blue) relative to the saw guide planning (green). **e** The reference box (black) is selected as the region of interest on the optical scan (red) to compare to the reference box of the planning (green) in order to calculate the transformation matrix *T* with eight corresponding placement errors. Relative to the planning position: Z-value increases when translating in proximal direction, Y-value increases when translating away from the bone, X-increases when translating to the left

The position of the placed sCT and CT based saw guides on the optical 3D scan were compared to the saw guide position on the planning [6]. The bone models from these optical 3D models were rigidly registered to the bones on the micro-CT in MATLAB (MathWorks, Natick, USA) with an iterative closest point (ICP) algorithm [20]. After registration with the ICP-algorithm, the displacement between the sCT or CT placement reference boxes and the corresponding planning reference box was calculated in a transformation matrix *T* (Fig. 6). From this matrix, eight displacement errors were determined: axis specific translational errors in the x, y and z-direction (∆x, ∆y, ∆z) and a total translation error ∆T = √((∆x)^2^ + (∆y)^2^ + (∆z)^2^) in mm and axis specific rotation errors around the x, y and z-axis (ϕx, ϕy, ϕz) and a total rotation ∆R = √((ϕx)^2^ + (ϕy)^2^ + (ϕz)^2^) in degrees [21].

### Statistics

Results were statistically analyzed with SPSS v.25 (IBM Corp, Armonk, NY, USA). A repeated measure analysis of variance (rANOVA) investigated between observers and between subjects differences in mean translation and rotation displacements of the sCT saw guides compared to the CT saw guides. Secondly, Bland-Altman plots of the ∆T and ∆R were created in order to assess to what extend sCT-based saw guide placement agreed with the CT-based saw guide placement. For this, two types of limits of agreement (LoA) were calculated and displayed: 1.96 × standard deviation (SD) of the intra- and inter-observer variability, with the inter-observer variability as the maximum difference. If 95% of the data of the ∆T and ∆R lies within the calculated LoA, the displacement errors of the CT and sCT-based saw guides where regarded equivalent. Thirdly, box plots were created to analyze differences between saw guide locations.

The hypothesis that mean absolute placement errors would be equivalent for both CT and sCT was tested against the alternative hypothesis of significantly different errors. We aimed to detect a true difference of a 1 mm translational or 2 degrees of rotational error [22] with a SD of 1 mm or 2 degrees [6] with 80% power at a significance level of 0.05. This resulted in a required sample size of 16 per group [23].

## Results

### Bone model comparison

**Fig. 7:**
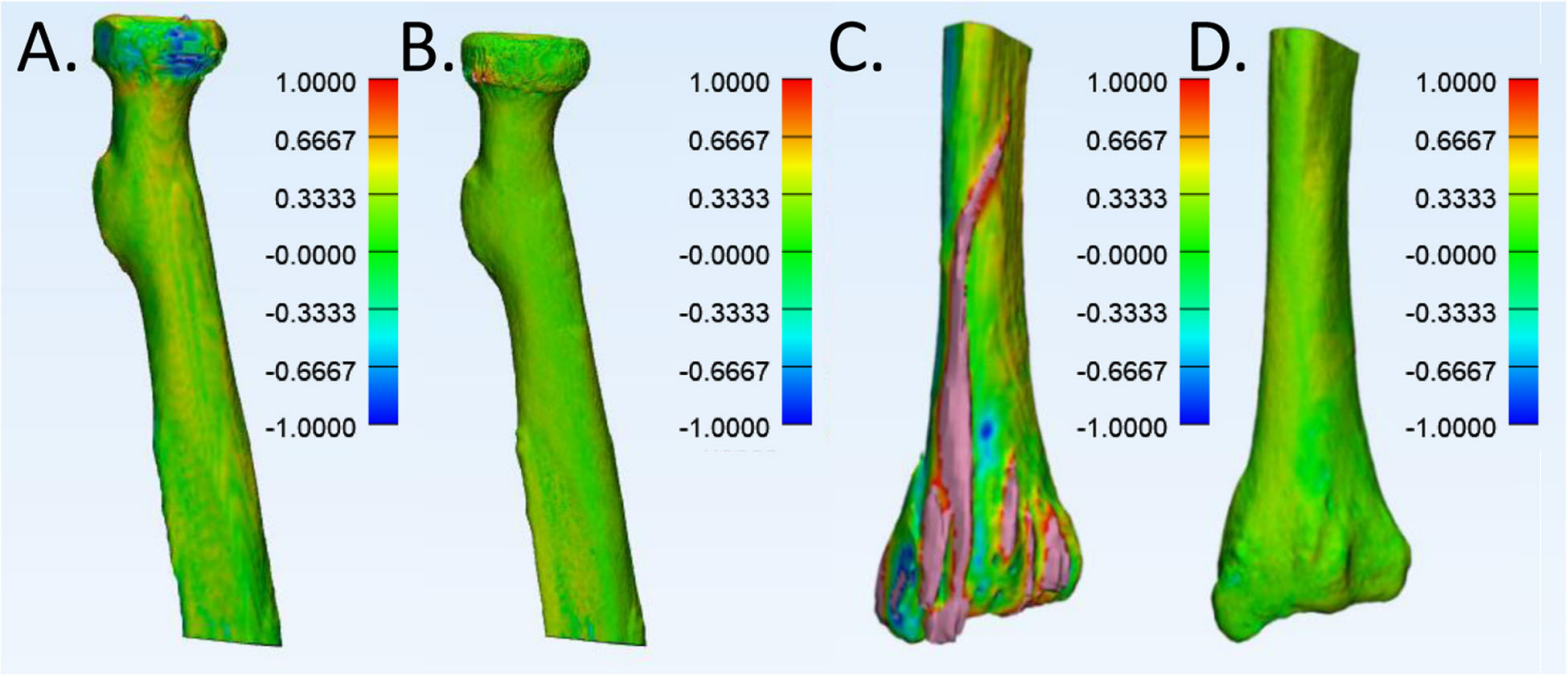
Distance mapping of surface model comparison. sCT (**a**) and CT (**b**) surface models compared to the micro-CT surface model of proximal radius. The color bar indicates differences (mm) between the micro-CT and the sCT or CT within a − 1 and 1 mm range. A positive value indicates a larger sCT or CT model than the micro-CT model. **c** Exhibits an inconsistency in the sCT surface model compared to the micro-CT surface model of a distal radius. The corresponding sCT saw guide was a placement outlier during the saw guide placement study. **d** Shows the CT surface model compared to the micro-CT surface model of the same distal radius

The average surface difference was 0.27 ± 0.30 mm between the micro-CT and sCT models and 0.24 ± 0.12 mm between the micro-CT and CT models. The positive number indicates a small overestimation of bone by the sCT and CT when compared to the ground truth micro-CT (Fig. 7). Differences were largest and most frequently seen near the joints at the proximal and distal bone ends of both CT and sCT (Fig. 7a,b). However, in one case, a sizable difference was found in the sCT surface model due to a false positive identification of a (calcified) tendon as bone (Fig. 7c,d).

### Influence bone simmering

The additional surface distance analysis between the pre- and post-simmering micro-CT showed an average surface difference of − 0.04 ± 0.12 mm indicating only a minimal shrinkage and therefore likely had a negligible effect on the study results (Appendix).

### Saw guide placement

**Fig. 8:**
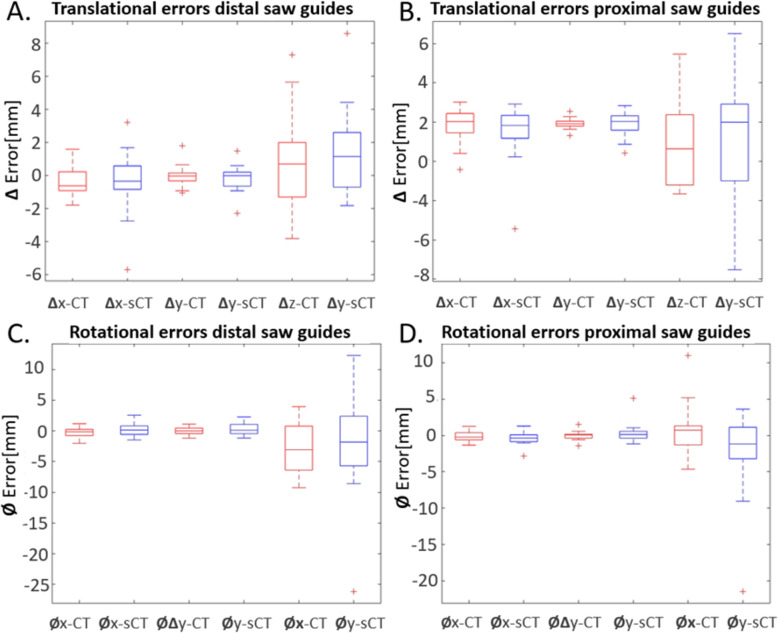
Box plots of **a** translation errors of distal guides, **b** translation errors proximal guides, **c** rotational errors of distal guides and **d** rotational errors proximal guides with a stratification for CT- (red) and sCT-based (blue) saw guide types. The central mark in the box indicates the mean, the top (Q3) and bottom (Q1) box edges are the 25th and 75th percentile. The whiskers extend to the most extreme data not considering outliers; outliers are defined as > 1.5 times the interquartile range and are marked with a red ‘+’ sign

Saw guide placement error for both sCT- and CT-based designs were assessed. The rANOVA showed no statistically significant difference in sCT and CT placement for all axis-specific translational and rotational errors as well for the ∆T and ∆R errors (Table 1). Additionally, no significant difference was found between observers. The average translation and rotation placement errors were the largest in the z-direction for both sCT and CT based designs (Fig. 8).

**Table 1:** Average (Standard deviation (SD)) absolute translation and rotation errors of the CT- and sCT-based saw guides placed by the six observers. The *p*-value was calculated with a rANOVA on the between subjects differences of each parameter, with *p* < 0.025 being significantly different

Saw guide type	Translation: mm (SD)					Rotation: degrees (SD)				
	∆x	∆y	∆z	∆T average	∆T max diff	ϕx	ϕy	ϕz	∆R average	∆R max diff
CT-based	0.8 (±1.1)	0.4 (±0.6)	2.1 (±2.3)	2.4 (±2.4)	4.5 (±3.4)	0.6 (±0.7)	0.5 (±0.6)	3.5 (±4.8)	3.8 (±4.8)	6.9 (±6.8)
sCT-based	1.0 (±1.3)	0.5 (±0.6)	2.3 (±2.4)	2.8 (±2.5)	4.5 (±2.8)	0.7 (±0.6)	0.8 (±0.7)	4.6 (±6.0)	4.9 (±6.0)	7.0 (±6.8)
*p*-value	.892	.687	.752	.284	–	.245	.167	.227	.216	–

**Fig. 9:**
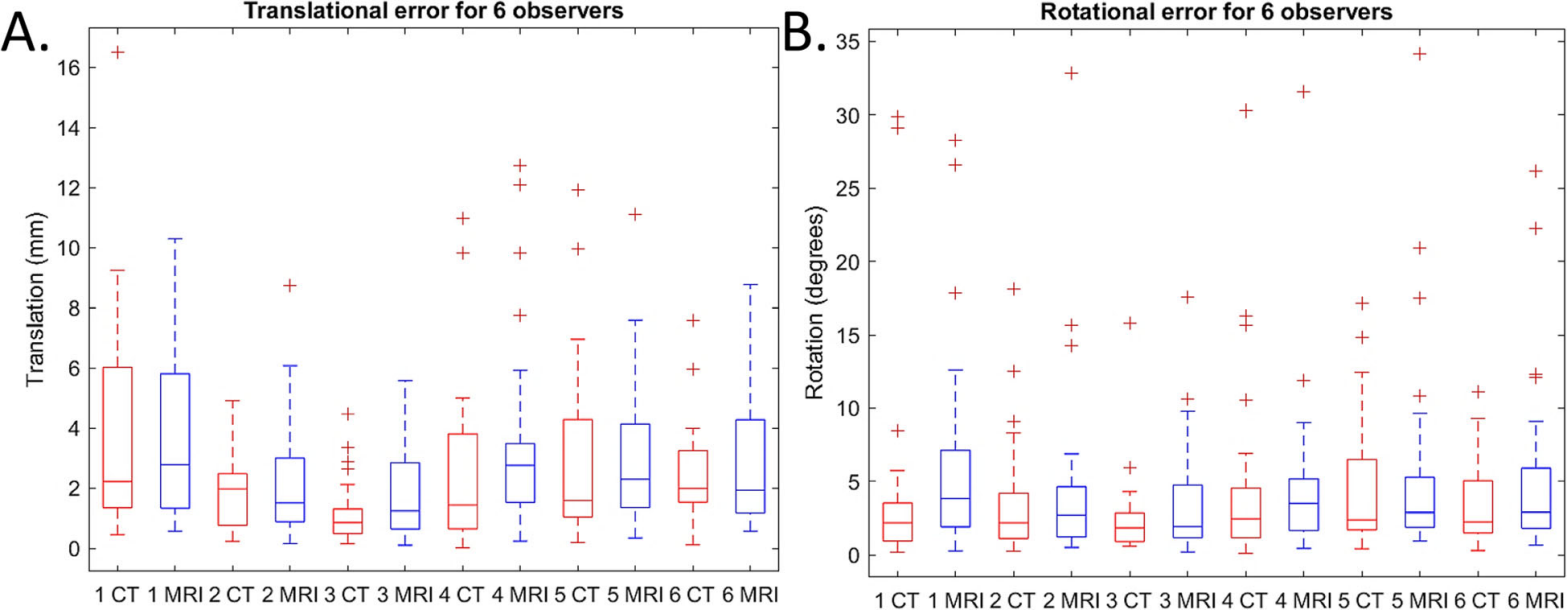
Box plots of the **a** total translation error ∆T and the **b** total translation error ∆R, stratified for the six observers and for CT based (red) or sCT-based (blue) saw guide design. The central mark in the box indicates the mean, the top (Q3) and bottom (Q1) box edges are the 25th and 75th percentile. The whiskers extend to the most extreme data not considering outliers; outliers are defined as > 1.5 times the interquartile range and are marked with a red ‘+’ sign

The placements errors ∆T and ∆R were stratified for individual observers (Table 2) and outliers were defined as values that were > 1.5 times the interquartile range of that observer (Fig. 9). Observer 1 and 4 are orthopedic surgeons, observers 5 and 6 are orthopedic surgeons in training and observer 2 and 3 are orthopedic researchers. In total 51 of the 768 (64 saw guides × 6 observers × 2 errors [∆T and ∆R]) placements errors were defined as outliers (7%). Of these 51 outliers 51% were CT, 49% were sCT, 33% were outliers on both CT and sCT within one location, 36% unique for CT and 31% unique for sCT, 78% were ulna saw guides, 71% were distal located ulna saw guides, 53% were rotational errors of distal located ulna saw guides.

**Table 2:** Average errors ∆T and ∆R (SD) of CT- and sCT-based saw guides found per observer

	Observer 1	Observer 2	Observer 3	Observer 4	Observer 5	Observer 6
∆T CT	3.9 (±3.5)	1.8 (±1.2)	1.1 (±1.0)	2.4 (±2.6)	2.9 (±2.9)	2.5 (±1.5)
∆T sCT	3.8 (±3.1)	2.1 (±1.9)	1.8 (±1.4)	3.4 (±3.1)	3.0 (±2.3)	2.8 (±2.2)
∆R CT	4.0 (±6.9)	3.6 (±3.9)	2.4 (±2.7)	4.4 (±6.1)	4.6 (±4.5)	3.4 (±2.8)
∆R sCT	6.0 (±6.8)	4.5 (±6.2)	3.8 (±4.0)	4.6 (±5.6)	5.4 (±7.0)	5.1 (±5.9)

**Fig. 10:**
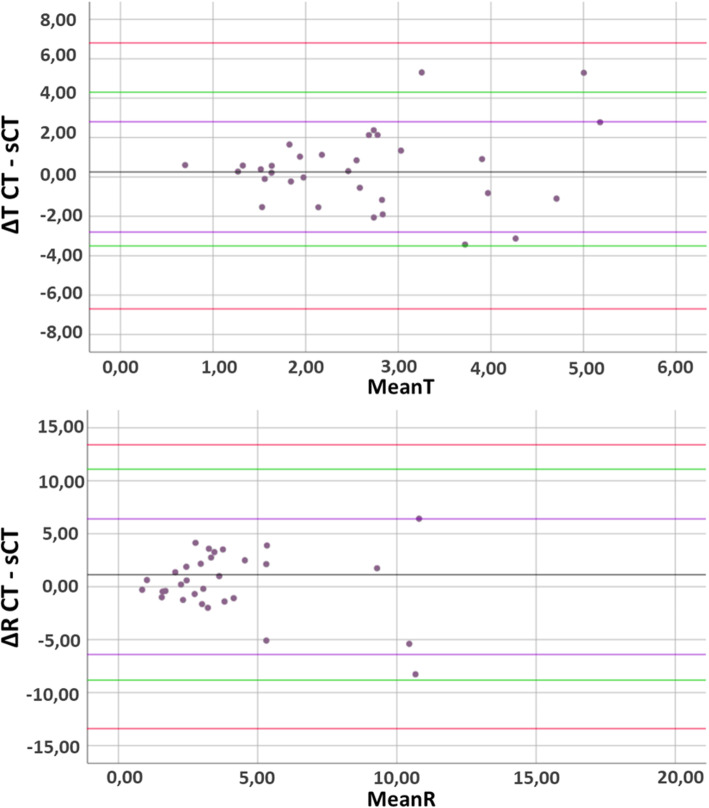
Bland-Altman plots. **a** The total translation error ∆T difference between CT- and sCT-based saw guides. The greyline represents the mean of 0.38 mm and **b** the total rotation error ∆R difference found between CT- and sCT-based saw guides with a mean of 1.13 degrees (grey line). Green lines display the 95% confidence interval of LoA 1.96 x SD. The purple and red lines are respectively the intra- and interobserver variability LoA (1.96 x SD)

Bland-Altman plots were computed containing the average differences between the CT and sCT-based saw guide ∆T and ∆R errors (Fig. 10). In addition to the standard LoA, the 1.96 × SD of the intra- and inter-observer variability limits of agreement are displayed. For both the ∆T and ∆R errors all values fall between the inter-observer LoA and almost all (30/32) values fall between the intra-observer LoA which was based on the best scoring observer #3 (Fig. 9).

## Discussion

The 3D model surface distance comparison showed similar errors for sCT and CT with respect to the ground truth micro-CT. The similarity between both imaging modalities was in line with the fact that no statistically significant differences were found when comparing all translational and all rotational saw guide placement errors of both modalities. Furthermore, Bland-Altman plots of the total rotation and total translation displacement showed that the LoA of these displacements were within the LoA of the inter-observer variability. These results indicate that the positioning errors of the CT- and sCT-based saw guides are comparable in the context of its relevance for clinical application.

In another publication on surgical guides design, Caiti et al. [6] analyzed positioning errors of distal, mid-shaft and proximal radius saw guides in vitro and also found the largest translation and rotation errors in the z-axis. Additionally, Caiti et al. showed that distal guides have the smallest total translation (ranged 0.25–1.8 mm) and rotation (ranged 0.2–1.6 degrees) errors when compared to proximal (respectively ranged 0.15–2.25 mm and 0.3–5.7 degrees) or mid-shaft guides (respectively ranged 0.4–3.2 mm and 1.3–7.3 degrees). These values are slightly smaller than the results of our current study, which might be explained by several aspects. First of all, Caiti et al. 3D-printed plastic radius bone models to place the saw guides on, while these models were also used to design the saw guides themselves. Our study used the actual cadaveric specimens. Secondly, different anatomical locations were used: Caiti et al. investigated three locations merely on the radius, while this study focused on two locations (distal and proximal) for both radius and ulna bones. In our study the distal ulna had the largest placement errors, which is probably due to the distal ulna being more circular shaped and largely anchorless. Thirdly, different saw guide lengths were used; the guides of Caiti et al. were slightly longer (> 50 mm) than our saw guides (40 mm), which may be expected to influence the stability as longer guide length have had more attachment anchors and thus result in smaller positioning errors. Note however that longer saw guides are not often clinically used [2]. Besides the differences, both studies used a simplistic design of the saw guides that allowed for high reproducibility but made them prone to placement errors on the mainly tubular shaped bones. Therefore, the placement accuracy of both sCT and CT generated saw guides might even better when used for actual clinical cases because the guides can be designed with a more three-dimensional fit accompanying specific surgical identifiable landmarks.

There are several study limitations and recommendations that should be noted. First, the initial resolution of the CT-scan was higher than the resolution of the MRI-scan used for sCT reconstruction: 0.3 versus 0.7 mm pixel spacing, 0.8 versus 1.2 mm slice thickness and 0.4 versus 0.6 mm spacing between slices. This relative high CT resolution and mAs was used to get a best case surface model, though is not often clinically used because of the radiation burden. Therefore, this could have positively influenced the accuracy of the CT-guides in comparison to the sCT-guides and may have caused the smaller standard deviation in the bone surface evaluation. However, despite these initial resolution differences, no statistically significant differences were found in saw guide placement. As a result, we can conclude that the MRI resolution was sufficient for sCT-based saw guide design and planning.

Furthermore, the currently used version for sCT generation may still contain inconsistencies [15]. Firstly, in one case, calcified tendons or vessels adjacent to the bone were falsely interpreted by the sCT model and presented as bone (Fig. 7c). However, no large average differences were found between sCT and CT placement errors for this bone, respectively ∆T of 1.0 mm versus 2.8 mm and ∆R 5.7 versus 4.0 degrees. Second, because the neural network is trained to generate the sCT with different data sets with each comprising a slightly different field of view, the network has the least (training) data on border visualizations, therefore the sCT sometimes delivers inhomogeneous densities near the distal or proximal bone ends (Fig. 7a,b) [15]. These inhomogeneities influenced the surface distance comparison, but should not have affected the accuracy of the saw guides as these were positioned further away from the joints (bone ends). Nevertheless, future research should focus on optimizing the sCT-algorithm with additional training data to further minimize false positive structures and errors in the sCT.

Another limitation is the difficulty of translating the results to clinically relevant outcomes for a lower arm osteotomy. The results in this study show displacement errors, where a larger displacement indicates a less accurate cutting plane compared to the planning. Ma et al. [22] showed the clinical relevance of distal radius osteotomy guides by translating the displacement errors to correction errors of the ulnar variance, radial inclination and volar tilt. A recommendation is to compare future results to those of Ma et al. by creating a virtual lower arm osteotomy model with the generated 3D models and translate the calculated displacement errors to clinical corrections. However, the main focus of this study was to assess the placement accuracy differences of the two different imaging modalities (MRI/sCT versus CT). For future use, the sCT scan should be validated with a sCT-based saw guide patient study.

Finally, this research on the accuracy of sCT generated saw guides for lower arm osteotomies sets an example for other areas with a high saw guide turnover (e.g. knee and craniomaxillofacial surgery) to also implement sCT. However, to get to the clinical application sCT training data should be acquired and validated in a similar fashion.

In conclusion, in this research we showed that sCT and CT provided similar digital models, as the surface distance with respect to the ground truth micro-CT was not significantly different in lower arms. Furthermore, the positioning of saw guides based on these sCT and CT models did not demonstrate significant differences in a cadaveric lower arm study and indicate that both methods are clinically equal. Therefore, a first important step is made in enabling radiation-less 3D planning and design of patient-specific saw surgical guides facilitated by MRI-based synthetic CT.

## Figures and Tables

**Fig. 11:**
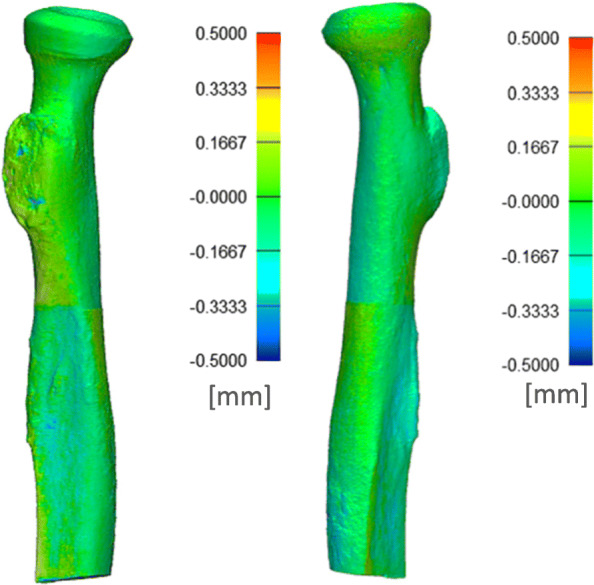
Distance mapping between the pre- and post-simmering micro-CT 3D model of a proximal radius of P1. The red indicates a positive distance value (volume increase) and blue a negative distance value (volume decrease) in the − 0.5 to 0.5 mm range

## Data Availability

The datasets used and/or analysed during the current study are available from the corresponding author on reasonable request.

## Appendix

### Simmering process

To increase the accuracy of the optical scanner to create a 3D bone surface model and to allow proper registration of this model to the micro-CT surface model, all soft tissues were removed prior to the saw guide placement after 12-h of simmering in physiological water.^20^

To analyze the influence of the simmering on bones, a second micro-CT on a random sub-selection of eight bones was made at the end of the study and compared to the micro-CT models before simmering. After rigid registration^17^ of the pre- and post-simmering models, the average distances between the 3D model vertices were calculated in mm in 3-matics (medical license version 13, Materialize NV, Leuven, Belgium). A positive value indicates a larger post-simmering 3D model than the pre-simmering 3D model.

The additional surface distance analysis between the pre- and post-simmering micro-CT showed an average surface difference of − 0.043 +/− 0.124 mm (mean +/− SD) indicating a minor shrinkage of the bones after simmering (Table 3, Figure 11). However the measured shrinkage was less than the 0.5 mm boiling shrinkage reported in literature^19^ and less then the 0.05 mm micro-CT resolution. Therefore, we expect that the simmering had minimal to no influence on the differences between the sCT and CT saw guides placement error, especially since the same bones were used for both modalities.

**Table 3:** Distance mapping of the microCT 3D model surface of eight halve bones before and after the saw guide study. Bone part 1 and 2, 3 and 4, 5 and 6 and 7 and 8 are from the same specimen

Bone part	1	2	3	4	5	6	7	8	Average
Mean [mm]	−0.037	−0.047	−0.036	−0.037	−0.028	−0.031	−0.065	−0.061	−0.043
SD [mm]	0.060	0.130	0.142	0.111	0.044	0.261	0.117	0.124	0.124

## Abbreviations

3D: 3-Dimensional
(s)CT: (synthetic) Computed Tomography
(c)GAN: (conditional) Generative Adversarial Network
HU: Hounsfield Units
ICP: Iiterative Closest Point
KV: Kilovoltage
LoA: Limits of Agreement
mAs: Milliampere-seconds
MRI: Magnetic Resonance Imaging
MR: Magnetic Resonance
MS: Millisieverts
rANOVA: Repeated Measure Analysis Of Variance
SD: Standard deviation
